# Magnitude, causes and characteristics of trauma victims visiting Emergency and Surgical Units of Dilchora Hospital, Eastern Ethiopia

**DOI:** 10.11604/pamj.2018.30.177.10969

**Published:** 2018-06-27

**Authors:** Lemma Negesa Bulto, Yadeta Dessie, Biftu Geda

**Affiliations:** 1 Haramaya University College of Health and Medical Sciences, School of Nursing and Midwifery, Harar, Ethiopia; 2 University of Adelaide, Adelaide Nursing School, South Australia, Australia; 3 Haramaya University Colleges of Health and Medical Sciences, School of Public Health, Harar, Ethiopia

**Keywords:** Trauma, causes of trauma, types of trauma, victim´s characteristics

## Abstract

**Introduction:**

In developing countries, approximately sixteen hundred people die every day from all types of injuries, making injury the third most common cause of all mortalities in the region. The objective of this study was to examine the magnitude, causes and types of traumatic injuries in patients visiting Dilchora Hospital in Dire Dawa Administration of Eastern Ethiopia.

**Methods:**

We conducted a cross-sectional study which was supplemented with qualitative data. Descriptive and thematic analyses were used to characterize the trauma in terms of victims, causes and types.

**Results:**

A total of 382 patients were identified within a two-month period of data collection. The most common causes of traumatic injuries identified were conflict (42.67%), road traffic accidents (35.07%), falls (13.35%) and burn injuries (8.90%). Soft tissue injuries (57.6%), abrasion (29.3%) and fractures (22.3%) were the three most common types of injuries experienced. Most of the victims were males, those people in the productive age bracket and those living in urban areas. Poor road structure, poor adherence to traffic rules and the use of old and poorly maintained vehicles were the main reasons for the road traffic accidents. Substance use behaviors in urban areas and farmland boundary issues in rural areas were the common causes of conflicts, and females were the most common victims of burn injuries.

**Conclusion:**

A high magnitude of injuries was observed such that, on average, six trauma victims visited the hospital each day. Conflict and road traffic accidents were the two most common causes of traumatic injuries. Poor vehicular conditions and poor road design were the main reasons identified for road traffic accident related trauma, which requires multilevel interventions.

## Introduction

Physical traumatic injury is physical harm to the human body which can result from accidents, falls, drowning, suicide, interpersonal violence, burns, poisoning and sports and work related accidents, but is not limited to these causes [1, 2]. Evidence has shown that about 5.8 million people die of traumatic injuries worldwide. Importantly, road traffic accidents (RTA) are the tenth leading cause of death and the ninth leading cause of the disease burden [3, 4]. Close to sixteen people die every day from all types of injuries in developing countries, making this the third most common cause of overall mortality, ahead of diarrhea, tuberculosis, and measles. Globally, an estimated 1.2 million people die from RTA each year, and as many as 50 million are injured. Statistical projections indicate this figure will increase by about 65% over the next 20 years unless there is a new commitment set for prevention [2, 5]. Developing countries are seriously impacted by such injuries, carrying 94% of the load, with injury the third leading cause of death ahead of diarrhea, tuberculosis, and measles [5]. The problem is no less severe in sub-Saharan Africa, where trauma is ranked as the third major cause of death and permanent disability among the adult population, next to tuberculosis and HIV/AIDS [5]. Ogendi and Ayisi (2011), in their Kenyan-based study, revealed that the leading causes of injuries were assault (42%), RTA (28%) and unspecified soft tissue injuries (STI) (11%). In addition, the study indicated falls and burns were infrequently reported (each < 10%) [6]. Similar findings from Cameroon showed RTA (56%), assault (22%) and domestic injuries (13%) were the leading causes of trauma [7]. Ethiopia is one of the countries in the region where injuries are a major cause of health problems that commonly appear as one of the top causes of morbidity and mortality [6]. Despite the prevalence of these catastrophic causes of health problems, there has been limited evidence about traumatic injuries in general and their respective characterization. The aim of this study was to assess the magnitude, causes and types of traumatic injuries in a referral hospital in Eastern Ethiopia.

## Methods

### Study setting

A cross-sectional study, supplemented with qualitative data, was undertaken from January to February 2013 in Dilchora Hospital, Dire Dawa Administration, Eastern Ethiopia. Dire Dawa Administration is located 515 kilometers from Addis Ababa, the capital city of the country. As per the 2007 census report, the population of Dire Dawa was 379,000 of which 70% reside in the urban areas. The majority of the population derived their livelihood through business activities, and the potential health coverage of the administration was 100%. Dilchora Hospital is the main government owned referral hospital in the administration [8] and provides a number of services, including emergency, outpatient and inpatient services.

### Study participants

All trauma cases that visited the Emergency Department, Surgical and Orthopedic in-patient Units of the hospital within the study period were recruited for the study. A total of 382 study participants, comprising of trauma patients, were included in the quantitative study. For the qualitative component, four focus group discussions (FGD) and fifteen in-depth interviews (IDI) were undertaken with a total of 15 residents, health care workers, security police and road traffic police were included in the qualitative component of the study.

### Measurements and data collection

Data collection tools were developed from a national injury recording format and injuries were characterized by socio-demographic characteristics, adherence to traffic rules and the use of intoxicants, including alcohol and khat, through an interview and patient self-report. The data collectors were trained for two days and the principal investigator monitored the data collection process on a daily basis. Qualitative data were collected through focus group discussions (FGD) and in-depth interviews (IDI) with residents, police and healthcare workers. Qualitative data were recorded in both cases. In this study, conflict is defined as fighting between two or more groups of people that results in harm to the body or injury, and a conflict victim is a person who is harmed in conflict as described above.

### Data analysis

The collected data were entered to Epi Data version 3.02 and exported to SPSS version 16 for analysis. Descriptive analyses that include proportion, percentage, and appropriate graphics presentations were applied to describe the data and to characterize trauma in relation to the causes and types of trauma, with socio-demographic characteristics used to describe the injury. Qualitative data were analyzed using Open Code through coding and recoding into themes addressing the different types of injuries.

### Ethical considerations

Ethical clearance was obtained from the Institutional Health Research Ethics Committee (IHREC) of Haramaya University, College of Health and Medical Sciences. Official permission letters were obtained from Dire Dawa Administration Health Bureau and Dilchora Hospital. Respondents were clearly informed about the purpose, risks, and benefits of the study and interviews were only conducted with those who gave written consent. The participants right to withdraw from the study at any stage was respected and names were not documented to maintain confidentiality.

## Results

### Magnitude and causes of traumatic injuries

A total of 382 trauma victims were identified over the two-month period. The major causes of injuries were conflict (163(42.67%)), RTA (134(35.07%)), falls (51(13.35%)) and burns (34 (8.90%)) (Table 1).

**Table 1:** Causes of traumatic injury among patients visiting, Dilchora Hospital, Dire Dawa, January-February,2013

Cause of injury	Total N (%)
Conflict	163(42.67%)
Road Traffic Accidents (RTA)	134(35.07%)
Falls	51(13.35%)
Fire	34(8.90%)
Total	382(100%)

### Characterization of the traumatic injuries

From a total of 382 trauma victims, 219 (57.33%) were unintentional and the rest 163 (42.7%) were intentional. Most conflict (84.66%) and RTA (76.86%) injures occurred among the 15-49 year age group, and conflict largely occurred among males (68.71%). Burn injuries were common among females (6.54%). The majority of RTA (67.91%) and violence (61.35%) victims were urban residents (Table 2). Of 382 victims, only two of them had a mental health problem while 13 had a history of seizures. Only six (1.6%) had a physical disability of whom one was deaf, three were blind and two had limb paralysis. From the total victims, 227 (59.4%) of them had good knowledge about road traffic rules while 155 (40.6%) had poor knowledge about traffic rules. Furthermore, 220 (57.6%) of them claimed that they had adhered to road traffic rules while 162 (42.4%) did not. From a total of 382 victims, 96 (25.1%) had a past history of conflict, 27.5% had chewed khat within six hours prior the incidents and 14.7% had consumed alcohol.

**Table 2:** Socio-demographic and behavioral characteristics of trauma victims among patients visiting Dilchora Hospital, Dire Dawa, January-February, 2013

Cause of trauma		RTC N (%)	Fall N (%)	Conflict N (%)	Burn N (%)	Total N (%)
Variables	Category					
**Sex**	Male	83(61.94%)	26(50.98%)	112(68.71%)	9(26.47%)	230(60.21%)
	Female	51(38.06%)	25(49.02%)	51(31.29%)	25(73.53%)	152(39.79%)
	Total	134(100%)	51(100%)	163(100%)	34(100%)	382(100%)
**Educational Status**	Illiterate	32(23.88%)	17(33.33%)	48(29.44%)	2(5.88%)	99(25.9%)
	Reading and writing only	9(6.72%)	3(5.88%)	4(2.45%)	4(11.76%)	20(5.24%)
	Grade 1-8	39(29.10%)	15(29.41%)	48(29.44%)	19(55.88%)	121(31.67%)
	Secondary school	28(20.90%)	14(27.45%)	43(26.38%)	8(23.53%)	93(24.3%)
	Diploma and above	26 (19.40%)	2(3.92%)	20(5.24%)	1(2.94%)	49(12.81%)
	Total	134(100%)	51(100%)	163(100%)	34(100%)	382(100%)
**Residence**	Urban	91(67.91%)	25(49.02%)	100(61.35%)	23(67.65%)	239(62.57%)
	Rural	43(32.09%)	26(50.98%)	63(38.65%)	11(32.35%)	143(37.43%)
	Total	134(100%)	51(100%)	163(100%)	34(100%)	382(100%)
**Occupation**	Government employee	25(18.66%)	3(5.88%)	15(9.20%)	1(2.94%)	44(11.52%)
	Merchant	13(6.70%)	1(1.96%)	14(8.58%)	0(0%)	28(7.33%)
	Daily laborer	4(2.98%)	5(9.80%)	12(7.36%)	0(0%)	21(5.50%)
	Private	22(16.42%)	10(19.60%)	39(23.92%)	3(8.82%)	74(19.37%)
	Student	34(25.37%)	12(23.52%)	38(9.95%)	23(67.65%)	107(28.01%)
	House wife	15(11.19%)	10(19.60%)	12(7.36%)	4(11.76%)	41(10.73%)
	Unemployed	8(5.97%)	6(11.76%)	12(7.36%)	1(2.94%)	19(3.97%)
	Farmers	13(9.70%)	4(7.84%)	21(12.88%)	2(5.88%)	40(10.47%)
	Total	134(100%)	51(100%)	163(100%)	34(100%)	382(100%)
**Age category**	<5	4(2.98%)	0(0%)	1(0.61%)	2(5.88%)	7(1.83%)
	5-14	14(10.45%)	9(17.64%)	11(6.74%)	19(55.88%)	53(13.87)
	15-49	103(76.86%)	32(62.74%)	138(84.66%)	13(38.23%)	286(74.87%)
	50-64	9(6.71%)	4(7.84%)	11(6.74%)	0(0%)	24(6.28%)
	65+	4(2.98%)	6(11.76%)	2(1.23%)	0(0%)	12(3.14%)
	Total **N** (%)	134(100%)	51(100%)	163(100%)	34(100%)	382(100%)

### Trauma injury types

Out of the total injuries, 220 (57.6%) were diagnosed with soft tissue injuries while 121 (29.3%) were diagnosed with abrasions and 85 (22.3%) were diagnosed with fractures. Most of the patients (164 (42.9%)) experienced head injuries, followed by injuries to lower extremities (124(32.4%)), upper extremities (102(26.7%)), and the thorax (31(8.11%)). Only 12 (3.14%) were diagnosed with abdominal injuries. A total of 253 (66.2%) were treated and sent home, 108 (28.3%) were admitted to the orthopedic or surgical wards, 12 (3.1%) were admitted to the Intensive Care Unit (ICU) and 9 (2.4%) were referred on to the next level institution for further investigation and treatment (Table 3).

**Table 3:** Types of injury and fractures among patients visiting Dilchora Hospital, Dire Dawa, January-February - 2013

Type of injury	Frequency	Percentage
Abrasion	121	29.3%
Avulsion	1	0.3%
Burn Wound	32	8.4%
Contusion	3	0.8%
Deformity	4	1%
Dislocation	40	10.5%
Ecchymosis	3	0.8%
Fractures	85	22.3%
Sprain	11	2.9%
Wound (soft tissue injuries)	220	57.6%
Total	520	131
**Bone fractured**		
Ulna	29	26.85%
Radius	12	11.1%
Humerus	4	3.7%
Femur	11	10.18%
Tibia	18	16.6%
Fibula	14	12.96%
Hip	6	5.5%
Rib	14	12.96%
Total	108	100

### Qualitative findings

Four Focus Group Discussions (FGD) were conducted: two with residents and two with health care workers. Fifteen in-depth interviews were undertaken with police officers, including security police and road traffic police. Several factors linked with traumatic injuries were identified from the data analysis under various themes as presented below.

### Road traffic accidents

#### Personal-related characteristics

Reasons given by the participants for road traffic accidents included poor knowledge of driving rules, poor adherence to traffic rules, substance use and under aged drivers. Regarding the drinking and khat chewing behaviors of young drivers, it was noted that many of them had undisciplined driving habits and usually exceeded the speed limit resulting in catastrophic accidents. One of the respondents stated:

“There are undisciplined drivers who do not regularly put on their seat-belts, do not give priority for pedestrians, even on ‘Zebra’ crossings (pedestrian crossings), drive drunk and chew ‘khat’, holding a cell phone and driving above the speed limit. They only adhere to traffic rules when they see traffic police, to escape from punishment”.

There is a huge problem with driver training in Ethiopia. The training is not up to the standard required, and the business of issuing driving licenses is often linked to corrupt practices. Many young people, who may even be under 18, lie about their age to be eligible for the license, which is legally issued after the age of 24 years. Furthermore, many driving licenses are issued without proper training. One of the participants mentioned that:

“As per the rules, the minimum age for public transport is 24 years, but very young individuals present false evidence that they are above 24 years of age and get licensed even at the age of 18 years or less. It is very common to see these young drivers driving at high speed, even in the town”.

In addition to driving-related factors, the community’s knowledge-related problems were also mentioned. The population has no adequate information and knowledge about the rules and regulations of safe driving. It is common to see many daily laborers or construction workers come to the town from the rural area in a utility vehicle or small truck loaded above capacity due to the requests from the passengers. Most of the time, there is no cooperation from the community to identify undisciplined drivers. As claimed by one of the respondents:

“It is surprising that many passengers support the drivers and ask the traffic police to excuse the driver rather than exposing the illegal acts of drivers”.

#### Vehicle-related characteristics

In Ethiopia, most of the commercial vehicles are old and second-hand cars are very common, which is a major contributor to road traffic accidents. A recent increase in the three wheeled ´Bajaj´ motor taxis has greatly contributed to increased traffic congestion and currently is responsible for a major share of the problems. One of the respondents said:

“The Bajaj taxis are travelling at high speeds and currently contribute significantly to road traffic accidents. As a solution, up to 20 to 30 Bajaj taxis can be substituted by single bus and decrease traffic on the road”.

#### Environmental factors

One of the main environmental factors identified as contributing to road traffic accidents was the road design and age. Along these routes, roads were constructed approximately 50 years ago and are damaged, very narrow, of defective design and with no separate pedestrian path. These problems are usually worsened by roadside objects. Design wise, road markings are usually invisible, are improperly placed or not replaced on time when they fade. There are often major and severe injuries and deaths on the narrow twisting roads like ‘Dangago’ to Dire Dawa. One of the interviewees expressed the following:

“Most of the roads are narrow and have no pedestrian walking way, and if there is a pedestrian walking way, it is used for marketing: such as selling hot breakfasts (“ful”) and other foods, so that the pedestrians will be forced to use the roads meant for the cars. As this happens when the traffic gets crowded in the morning and lunchtime, it largely contributes to road traffic accidents”.

### Conflict victims

Individual-level factors such as being violent, aggressive, poor behavioral control and negative social attitudes were raised as being causes of injuries emanating from conflict. Most importantly, the health care workers reported that the authoritarian childrearing attitudes, low parental education and income, parental substance use, poor parental monitoring of children and poor family functioning are associated with the occurrence of violence. There is no single risk factor but a combination of factors which predict violence occurrence and result in conflict inflicted injuries. One of the respondents reported that:

“The main risk factors for violence in adolescence include involvement in serious criminal acts and substance use, puberty, being male, aggressiveness, low family socioeconomic status and antisocial parents".

In the eastern part of the country, one prominent factor which contributes to conflict related injury is the living conditions of the population. In this part of the country, many of the residents live in an agrarian society where they are primarily dependent on farmland. However, because of their population size, the land allocation size is very limited.

“In rural areas, conflict (‘Mencha Injury’) among farmers over farmland and water are common causes of violence and conflicts".

### Burn injuries

Most health care workers reported that gender and individual lifestyle are associated with burn injuries. Rural residents always use open fire to prepare food and for light. This gives them a higher risk of experiencing burns. Electric corporation employees also commonly encounter electric burns resulting from their work. One of the individuals mentioned that:

"Individuals, especially females who use ‘Butagaz´ (gas stoves) and firewood to prepare food commonly encounter burn injuries from the nature of their work”.

### Fall injuries

A number of healthcare workers opined that age, gender, work conditions and place of residence were the main factors linked with falls. Aged people are more likely to experience falls due to weakness. Farmers and females were also reported as more likely to experience falls. More recently, linked to the increase in constructions, those who work in construction are also becoming victims of such injuries. One of the respondents reported:

“There is an increasing trend of construction activities in the nearby towns where many daily laborers are working. Many of these workers experience fall injuries”.

## Discussion

Our findings identified that the magnitude of trauma injury was high. Conflict and road traffic accidents were the main causes of injuries in the study area. This is consistent with a similar study conducted in Kenya by Ogendi and Ayisis (2011), which found that assault related injury (42%) followed by RTA (28%) were the prevalent problems [9]. Furthermore, a study from Cameroon also identified RTA and assault as the top two causes of trauma [7]. In Ethiopia, likewise, studies in Addis Ababa and Gondar indicated RTA is the most common cause of trauma followed by conflict [6, 10]. The fact that conflict was more prevalent than RTA in the current study might be attributed to the high prevalence of substance abuse, mainly ´khat,´ which causes people to have lower tolerance and more aggression. The other probable reason for high levels of personal conflict is the scarcity of arable land for individual farms. In the study area, there is a high population density in relation to the farmland area available, which frequently causes conflicts and fights.

The findings also revealed that burn injuries are more common among females (6.54%) compared to their counter parts (2.35%). This could be because females are mainly involved in kitchen activities such as cooking and other activities which involve fire. In line with this, a study from Mekele town, Ethiopia, revealed the annual burn incidence was high, and more common among females [11].

Fall-related injuries contributed to 13.35% of the traumatic injuries and were common among the aged, overweight and female populations. In line with this, a study from Northwest Gonder, Ethiopia, found that falls contributed to 18% of trauma cases [6]. Data from the Centers for Disease Control and Prevention also consolidate this finding: close to 32 percent of community dwelling individuals over the age of 65 fall each year, and females fall more frequently than males in this age group [12].

This study revealed that 74.84% of the victims were between 15-49 years, with males constituting 60.2%. This is supported by several studies, such as a study from Kenya where (66%) of trauma victims visiting the hospital were male [9], a study conducted in Black Lion Hospital in Addis Ababa, Ethiopia where 72.5% of trauma victims were within the ages of 15-44 years, of whom 73.2% were male [10] and a study from Jimma, Ethiopia with the same findings [11]. It is possible to predict that the number of injuries among working-aged men has a serious impact on the socio-economic development of the community. Further implications can be deduced as in Ethiopia, most of the households are headed by males, which will then impact on the income of families and communities.

Individual-related behavioral factors such as substance use, family-related factors such as social status and environmental factors such as land scarcity were identified by the community as being associated with the different injuries. These factors are consistent with global research reports explored by the World Health Organization (WHO) which state that individual factors, including being a victim of violence, low educational level, a history of aggression, substance use, proximal social relationships, and socio cultural factors significantly influence the levels of different health related injuries [13].

Driver errors including drunk driving, khat chewing, drug addiction and driving at excess speed were the main determinants of RTA. In addition, pedestrian road users’ lack of traffic rule awareness, poor vehicle status and narrow and damaged roads in the city contribute to RTA. Along with this finding, data from a WHO report indicates the underlying reasons for road crashes were improper drivers or low skill drivers resulting in drivers not giving priority to pedestrians, poor vehicle technical condition, pedestrians not taking proper precautions, and safety considerations not sufficiently prioritised in road development [14]. Excessive alcohol intake and addictions reduce the concentration and motor control of individuals which exacerbate the problem [15, 16]. Furthermore, poor road networks, poor knowledge of road traffic safety, low legislation enforcement, poor conditions of vehicles largely contribute to such problems [14].

The study is not without limitations that need to be mentioned. In particular, the respondents’ level of anxiety and fear of medico-legal cases might constrain the sincerity of the respondents when reporting on substance-use and affect the amounts and the quality of information. The use of cross-sectional study may not establish cause and effect relationship.

## Conclusion

The number of trauma patients was high, with an average of six victims per day, and conflict and RTA were the two most common causes of injury. The most productive age groups were more affected by trauma from conflict and road traffic accidents, and most of the reasons identified for trauma are easily preventable through education and infrastructure improvement. Interventions that focus on drivers’ risky behavior, speeding and road safety improvements are essential to avert trauma caused by RTA. Furthermore, the results of the study confirm that there is high need for a trauma treatment center to be established separately from the other departments, to provide service for trauma patients visiting the facilities. Furthermore, more research is needed to identify the most effective interventions to address the different types of injuries.

### What is known about this topic

- The burden of trauma from different causes is high and is becoming one of top five causes of mortality and morbidity.

### What this study adds

- Conflict is the most common cause of trauma followed by RTA in the study area;
- Drivers’ risky behavior and poor road networks are associated with RTA;
- Conflict is predominant in the area related to the farmland ownership and boundary issues in rural areas and substance abuse in urban areas.

## Competing interests

The authors declare no competing interests.
